# Longitudinal GluCEST MRI Changes and Cerebral Blood Flow in 5xFAD Mice

**DOI:** 10.1155/2020/8831936

**Published:** 2020-11-25

**Authors:** Hironaka Igarashi, Satoshi Ueki, Hiroki Kitaura, Tae Kera, Ken Ohno, Masaki Ohkubo, Mika Terumitsu-Tsujita, Akiyoshi Kakita, Ingrid L Kwee

**Affiliations:** ^1^Center for Integrated Human Brain Science, Brain Research Institute, University of Niigata, Niigata, Japan; ^2^Department of Pathology, Brain Research Institute, University of Niigata, Niigata, Japan; ^3^Department of Radiological Technology, School of Health Sciences, Faculty of Medicine, University of Niigata, Niigata, Japan; ^4^Neurology, University of California, Davis, USA

## Abstract

Many of the focal neurological symptoms associated with Alzheimer's disease (AD) are due to synaptic loss. Glutamate chemical exchange saturation transfer (GluCEST) magnetic resonance imaging (MRI) is a candidate method to assess synaptic dysfunction. We assessed chronological changes in GluCEST in a 5xFAD mouse model of AD, comparing Glucest effects and regional cerebral blood flow (CBF). GluCEST effects and CBF in 5xFAD mice aged 1–15 months and their littermates (WT) were measured. Neurite orientation dispersion and density imaging (NODDI) MRI reflecting dendritic/axonal density was also measured and compared with GluCEST in 7-month-old mice. While regional CBF's decrease began at 7 months, GluCEST-reduction effects preceded hypoperfusion of the temporal cortex and hippocampus. While longitudinal 5xFAD mouse measurements revealed a correlation between the regional GluCEST effects and CBF, a generalized linear mixed model revealed statistically different correlations in cortical and basal brain regions. Further, NODDI-derived neurite density correlated with GluCEST effects in the parietal cortex, but not in the hippocampus, thereby revealing regional differences in pathophysiological mechanisms. Finally, GluCEST's effects correlated with regional synaptophysin. These results demonstrate that GluCEST can reflect subtle synaptic changes and may be a potential imaging method for AD diagnosis as well as serve as a biomarker of AD progression.

## 1. Introduction

Glutamate is the major neurotransmitter in the brain. Its central neuronal function is thought to be related to synaptic transmission [1, 2]. In brain circuits, glutamatergic synapses play a major role in brain functions, such as cognition, hampered in Alzheimer's disease (AD) [3]. At the core of the pathological changes of many neurodegenerative diseases is the accumulation of abnormal proteins, such as amyloid-*β* (*Aβ*) and tau in AD [4]. However, there is evidence that synaptic dysregulation reflects more directly the cognitive dysfunction in AD than the accumulation of abnormal proteins [5–7]. An examination of postmortem brain pathology in the nun study, which tracked the cognitive function of monastic nuns over a long period, beginning in the United States in 1986, revealed that about 30% of cases with AD had normal cognitive function up until immediately before death, thought to be due to their high “cognitive reserve” [8]. Supporting this report, spine density, a component of glutamatergic synapses, was maintained in Brodmann area 46, an area reportedly responsible for memory retention, in older adults presenting AD with normal cognitive function. However, spine density was reduced in those with cognitive decline [9]. These reports suggested that local synaptic density in the brain reflects various neural functions. Since about 80% of synapses in the mammalian brain are glutamatergic [10], we hypothesized that the quantification of brain glutamate may be used to assess synaptic density in normal and pathological brains, where the glutamatergic biosynthetic cycle is relatively stable and does not undergo any rapid metabolic changes. To verify this hypothesis, we demonstrated that a decrease in synaptic density in the cerebral cortex with synaptic pruning during normal growth from childhood to adulthood could be detected *in vivo* by proton magnetic resonance spectroscopy (MRS) to quantify glutamate concentration [11]. We also found that in the multiple system atrophy predominating in cerebellar ataxia (MSA-C), and where neurogenic respiratory failure is a major cause of death, pontine glutamate concentrations were correlated with respiratory function, but pontine volume from morphological images was not [12].

Proton MRS is broadly utilized to assess metabolic changes in early or prodromal AD stages [13, 14], since biochemical changes in AD are thought to precede the structural changes used in daily clinical settings [15]. Several reports have documented a focal glutamate decrease in the AD brain or AD animal models [16, 17]. However, the inherently low spatial resolution of MRS prevents the visualization of glutamate distribution in the brain. Glutamate chemical exchange saturation transfer (GluCEST) magnetic resonance imaging (MRI) [18], based on the exchange between the saturated amine protons of glutamate and bulk water, is a promising technique aiming to address this issue. To summarize briefly, saturated amine protons by radiofrequency pulse are transferred to water protons and lead to a decreased water proton signal depending on exchange speed and glutamate concentration. Targeting exchanging water protons enhances the detecting sensitivity of glutamate and enables high-resolution imaging. This technique has been applied to neurodegenerative diseases such as AD mouse models [19], frontotemporal dementia, and parkinsonism linked to chromosome 17 [20], Parkinson's disease [21, 22], and Huntington's disease [23].

To investigate chronological changes of GluCEST effect and their correlation with synaptic and/or neuronal degenerations, we employed GluCEST to detect chronological changes in 5xFAD AD mice [24] and compared them with their littermates (WT). 5xFAD mice show amyloid beta (*Aβ*) deposition as early as 2 months and impairment of spatial working memory by 4 to 5 months. Since thus far no other study has assessed GluCEST chronological changes in AD mouse model, we chose this model for its aggressive and distinct phenotype to investigate GluCEST chronological changes in disease progression and correlated with synaptophysin concentrations, which are considered to reflect synaptic density [25]. We also compared the focal chronological relationships between GluCEST effects and cerebral blood flow (CBF), regarded as a standard diagnostic biomarker in clinical settings, and the intracellular volume fraction (Ficvf), and considered to reflect axonal and dendritic density in the brain, using neurite orientation dispersion and density imaging (NODDI) [26].

## 2. Material and Methods

### 2.1. Experimental Animals

This study was approved by the Institutional Animal Care and Use Committee of the University of Niigata (SA00134) and conducted in accordance with the US National Institutes of Health guidelines regarding the care and use of animals for experimental procedures [27]. This study was performed on 5xFAD transgenic mice expressing five mutant human genes associated with AD, i.e., three amyloid precursor protein (APP) genes (*APPswe*, *APPflo*, and *APPlon*) and two presenilin 1 (PS1 and PSEN1) genes (*PSEN1 M146L* and *PSEN1 L286V*) [24], and WT mice. Breeding progenitors were purchased from The Jackson Laboratory (Bar Harbor, ME, USA). All animals were maintained under standard laboratory conditions with a 12 h/12 h light/dark cycle. Food and water were available ad libitum. Genotypes of all mice were determined by polymerase chain reaction analysis of DNA obtained by tail biopsies. The 5xFAD mice and their WT littermates were imaged at successive time points, in three cohorts. In cohort 1, 5xFAD mice (*n* = 11) and WT (*n* = 11) were imaged at 1, 4, 7, 10, and 15 months of age for CBF and GluCEST images at caudate slice. In cohort 2, 5xFAD mice (*n* = 12) and WT (*n* = 12) were imaged at 1, 4, and 7 months of age for CBF and GluCEST images at hippocampus slice, and immediately after the last MRI, brains were harvested to correlate MRI to pathological and biochemical inspections. In cohort 3, 5xFAD mice (*n* = 6) and WT (*n* = 6) were imaged at 10 and 15 months of age for CBF and GluCEST images at hippocampus slice. Some data were discarded due to excessive animal motion compromising image quality. Since the survival rate of 5xFAD mice dropped after >10 months of age [28], only nine 15-month-old survivors (5xFAD *n* = 4, WT *n* = 5) were imaged.

### 2.2. MRI Preparation and Measurement Protocol

MRI was performed using a 15 cm bore 7-T horizontal magnet (Magnex Scientific, Abingdon, UK) with an Agilent Unity-INOVA-300 system (Agilent Inc., Palo Alto, CA, USA) equipped with an actively shielded gradient. A custom-made volume transmitter and quadrature surface receiver proton coil (Takashima Seisaku-Syo, Hino, Japan) were used for MRI measurements. Mice were anesthetized using isoflurane (induction at 3–4%, 1.2–1.5% maintenance) mixed with 30 : 70 oxygen: N_2_O at 2 L/min and placed into the magnetized space. Mice were secured to a custom-made Plexiglas stereotactic holder. The head was fixed in place at the level of the ear using tooth bars. The rectal temperature was maintained at 37 ± 0.5°C using a custom-designed temperature control air-conditioning system.

The imaging protocol was carried out in the following order: (1) localizers (three planes), (2) T2-weighted fast spin-echo morphological images, (3) GluCEST, and (4) CBF imaging. The total acquisition time for each mouse was 121 min. For 7-month-old mice, additional diffusion-weighted images (3 shells, 109 scans/images, acquisition time 22 min) were obtained to assess regional differences regarding axon/dendrite density and GluCEST, since 7-month-old 5xFAD mice are reported to show neuronal loss in layer 5 in the cerebral cortex but not in the hippocampus [29].

### 2.3. MRI Acquisition and Image Postprocessing

#### 2.3.1. GluCEST Imaging

GluCEST imaging constituted of an 800 ms-long saturation pulse train (consisting of a series of 99 ms Hanning windowed saturation pulses with a 1-ms interpulse delay (eight 100 ms pulse train)) at B1rms = 5 *μ*T followed by the acquisition of a centric ordered snapshot fast low angle shot (FLASH) image. Imaging parameters were as follows: slice thickness = 1 mm, flip angle = 10°, matrix = 128 × 64, TR = 4.2 ms, TE = 1.9 ms, shot TR = 8,000 ms, and averages = 2. Raw CEST images were acquired at varying saturation offset frequencies, from −5 ppm to 5 ppm regarding water peak, with a step-size of 60 Hz. To map B0 inhomogeneity, water saturation shift referencing images with 100 ms Hanning windowed saturation pulses at B1rms = 0.1 *μ*T were acquired at saturation offset frequencies ranging from −0.5 ppm to 0.5 ppm regarding water peak, with a step-size of 6 Hz. The acquisition procedure was repeated two times to obtain images of the caudate and hippocampus.

The GluCEST MRI can map high-resolution glutamate brain distribution using the exchange between magnetically-labeled glutamatergic endogenous amine protons and water protons [18]. Regarding saturating glutamatergic amine protons at 3 ppm offset from water, the exchange of water between glutamate and water protons reduces the water signal. The reduction in water signal is quantified as the asymmetry ratio between images acquired with saturation at the resonant frequency of glutamatergic amine protons (3 ppm offset from water) and mirror saturation frequency regarding water (−3 ppm), which is equal to GluCEST = (M_−3ppm_-M_+3ppm_)/M_−3ppm_, where M_−3ppm_ and M_+3ppm_ are B0-corrected images saturated at −3 ppm and +3 ppm, respectively. CEST images obtained from −5 to 5 ppm were interpolated using the cubic spline method to generate images with a step-size of 0.05 ppm, and B0 inhomogeneity was corrected pixel by pixel using a modified MATLAB script [24] with WASSR data acquisition [30]. Six regions of interest (ROIs), namely, the frontal, parietal, and temporal cortex, caudate, hippocampus, and thalamus were manually chosen for numerical data analyses according to anatomical structures obtained from morphological images. The same ROIs were applied to the imaging methods described later.

#### 2.3.2. Cerebral Blood Flow Imaging

Cerebral blood flow was measured with continuous spin labeling (CASL) [31] following centric ordered snapshot-FLASH (with acquisition parameters identical to those for GluCEST). A 3 sec rectangular pulse at  ±10 mm from the imaging slice was alternatively irradiated with a 1 G/cm axial gradient for 0.8 sec before FLASH acquisition. Sixty-four image pairs were summed to enhance the signal-to-noise ratio. The magnetization transfer ratio was evaluated under the same conditions as the cerebral perfusion measurements, but without an axial gradient for adiabatic inversion. T1 measurements were performed using a centric ordered snapshot-FLASH with a hyperbolic secant inversion pulse and a 32-point (20 to 5000 msec) inversion delay [32]. Quantitative CBF maps were calculated from the cerebral perfusion image, T1 map, and magnetization transfer map according to the theory described by Ewing et al. [33] Maps were computed using a specialized MRI image calculation software (MR vision, MR vision co., Menro Park, CA, USA).

#### 2.3.3. Neurite Orientation Dispersion and Density Imaging

In 7-month-old mice measured CBF and GluCEST images at hippocampus slice, diffusion-weighted images (DWIs) were obtained to assess the regional relationship between axon/dendrite density and GluCEST effects. Axonal/dendritic density maps were computed as intracellular volume fractions (Ficvfs) from multiple raw DWIs according to the NODDI model [21], which enables the quantification of neurite density with an orientation-dispersed cylinder model and Watson distribution. DWIs were obtained with four shots spin-echo echo-planar imaging (EPI) sequences constituting three *b* values (300, 800, and 2500 s/mm^2^) acquired along 9, 27, and 62 directions of diffusion gradients, respectively. Each DWI acquisition was complemented with a gradient-free image (*b* = 0). The acquisition parameters were as follows: TR, 1350 ms; TE, 26.5 ms; diffusion gradient pulse duration (*δ*), 8 ms; diffusion gradient separation (Δ), 20 ms; field of view, 20 × 20 mm; matrix size, 128 × 64; slice thickness, 1 mm with 15 contagious slices. The obtained images were corrected for eddy-current distortion using DSI studio (http://dsi-studio.labsolver.org) [34]. All Ficvfs images were acquired according to the NODDI model and computed using the NODDI MATLAB Toolbox (http://www.nitrc.org/projects/noddi_toolbox).

### 2.4. Microscopic Inspection and Synaptophysin Measurements

To assess neuronal density in the hippocampus, where neuronal loss is not expected in 7-month-old 5xFAD mice, formalin-fixed hemi-brains were coronally sliced at the hippocampus level and paraffin-embedded. Then, paraffin-embedded brain blocks were sectioned in 4 *μ*m thickness and Nissl staining was performed. The image was acquired with CCD camera (MICROSCOPYDP73, Olympus, Tokyo, Japan) equipped with a microscope (BX53; UPlanSApo x40, NA: 0.95, Olympus).

Synaptophysin, a synaptic vesicle glycoprotein, was used to measure synaptic density by ELISA according to the method by Oakey et al. [24]. The frozen hemi-brain was homogenated using 1% Triton X-100, dissolved in PBS with 1 : 1 protease and phosphatase inhibitor cocktail. Consequently, synaptophysin was quantified using Mouse SYP/Synaptophysin ELISA Kit (LSBio, Seattle, WA, USA).

### 2.5. Statistical Analyses

SPSS version 25 (IBM Corp., Armonk, NY, USA, RRID: SCR_002865) was used for two-way analyses of variance (ANOVAs) to assess differences in the chronological progression of numerical data between different genotypes (WT vs 5xFAD). Tukey's honestly significant difference test was also used to compare the statistical differences between different time points for each genotype. Correlations between GluCEST effects and CBF were calculated using Sigmaplot Ver.12 (Sistat Inc. San Jose, CA, USA, RRID: SCR_003210).

R version 3.6.3 (R Foundation for Statistical Computing, Vienna, Austria; RRID: SCR_001905) was used to compare the correlation states between the GluCEST effects and CBF in each region using a generalized linear mixed model (GLMM) [35].

Graphpad Prism (Graphpad Software, San Diego, CA, USA RRID: SCR_002798) was used for unpaired Student's *t*-tests to compare numerical data between 5xFAD mice and littermates to assess regional differences in Ficvf in 7-month-old mice.

A *p* < 0.05 was considered statistically significant. All data are shown as mean ± standard deviation.

## 3. Results

### 3.1. Regional GluCEST Effect

**Figure 1:**
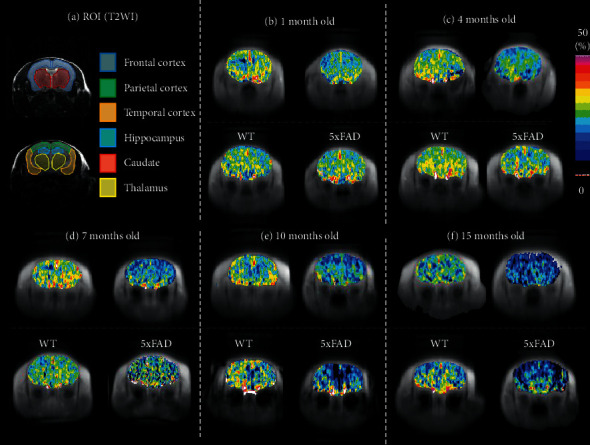
GluCEST MRI of 5xFAD and WT mice. (a) T2-weighted images (T2WI) showing ROIs in coronal brain slices. (b–f) GluCEST MRI of the corresponding T2WI showing reduced GluCEST effects in an aged 5xFAD mouse.

**Figure 2:**
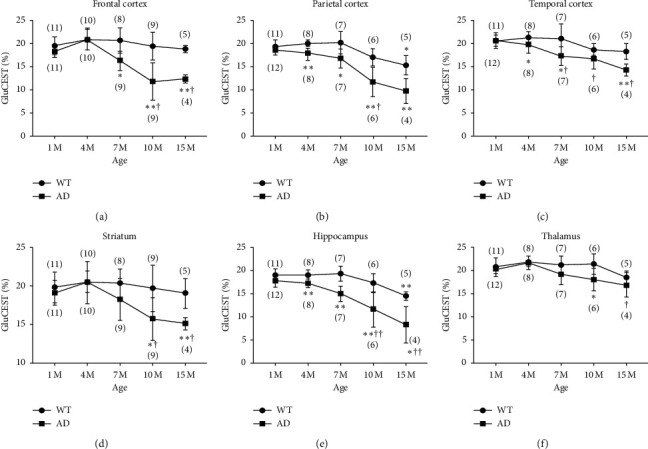
Chronological changes of GluCEST effects in 5xFAD and WT mice in the same ROIs as in T2WI images from Figure 1. GluCEST effect was significant at 4 months of age (parietal and temporal cortex, hippocampus) to 7 months of age (frontal cortex), except in the striatum and thalamus, where a decrease was observed by 10 months of age. In WT mice, a focal decrease (parietal cortex and hippocampus) in GluCEST effects became prominent only at 15 months of age. The number in parentheses indicates the number of analyzed mice. ^*∗*^*p* > 0.05, ^*∗∗*^*p* > 0.01 compared with 1-month-old mice. ^†^*p* > 0.05, ^††^*p* > 0.01 compared with WT mice.

Representative GluCEST maps of 5xFAD and WT mice are shown in Figure 1. Chronological changes in numerical values in each ROI of 5xFAD mice showed an earlier global decrease compared to WT mice, with these differences increasing with age (Figure 2). In all regions of 5xFAD mice, the decrease in GluCEST effects became statistically significant at around 1 and 4 months of age (parietal and temporal cortex, hippocampus) to 7 months of age (frontal cortex), except for the striatum and thalamus, where a decrease was observed only at 10 months old. In WT mice, the focal decrease (parietal cortex and hippocampus) in GluCEST effects only became prominent in 15-month-old mice probably due to senile decay; however, these reductions were smaller than those seen in AD mice.

### 3.2. Regional Cerebral Blood Flow

**Figure 3:**
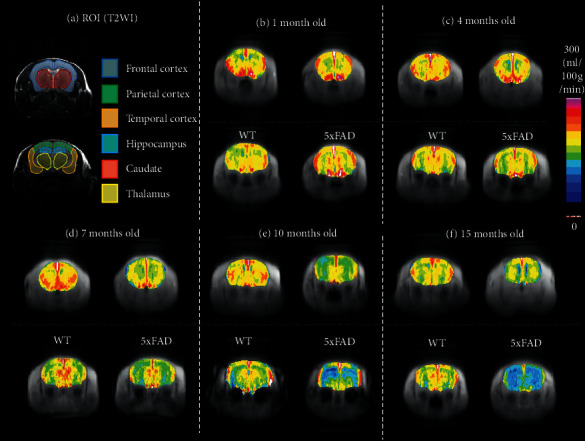
CBF of 5xFAD and WT mice. (a) T2-weighted images (T2WI) showing ROIs in coronal brain slices. (b∼f) CBF of corresponding T2WI showing reduced CBF in aged 5xFAD mouse. Units: ml/100 g/min.

**Figure 4:**
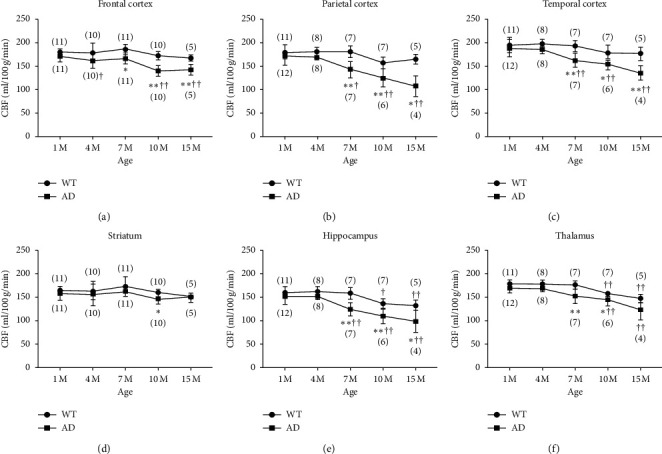
Chronological changes in CBF of 5xFAD and WT mice in corresponding ROIs as those in T2WI images in Figure 3. CBF in all regions except for the striatum significantly decreases in 5xFAD mice by 7 months of age. In WT mice, CBF in the hippocampus and thalamus decreased by 10 months of age, whereas all other ROIs had identical values throughout the study. The number in parentheses indicates the number of analyzed mice. ^*∗*^*p* > 0.05, ^*∗∗*^*p* > 0.01 compared with 1-month-old mice. ^†^*p* > 0.05, ^††^*p* > 0.01 compared with WT mice.

Representative CBF maps (Figure 3) and chronological CBF changes in ROIs (Figure 4) showed global CBF decreases, except in the striatum, in 5xFAD mice by 7 months of age. As observed from the GluCEST effects, age-dependent CBF decreases in 5xFAD mice are more severe than those in WT mice. In these mice, hippocampal and thalamic CBF decreased by 10 months of age, whereas all other ROIs showed identical values throughout the study.

### 3.3. Correlation between GluCEST Effects and CBF

**Figure 5:**
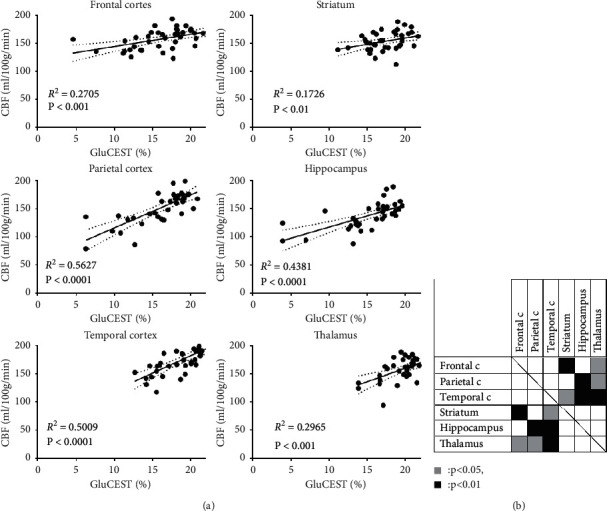
Correlation between GluCEST effects and CBF. (a) CBF of 5xFAD mice in all regions were statistically correlated with GluCEST effects. (b) GLMM revealed that correlations in cortices were statistically different from those of basal brain regions, except for the frontal cortex-hippocampus and parietal cortex-striatum.

CBF of 5xFAD mice in all regions was correlated with GluCEST effects (Figure 5(a)). Since part of the correlational mechanisms in cortices and other basal regions are thought to be different due to differences in anatomical and neural circuit structures, a generalized linear mixed model (GLMM) was used to assess differences in the correlations (Figure 5(b)). Considering random effects on both the intercept and the correlation line's slope, different correlational behaviors were observed in between cortices and basal regions, except in the frontal cortex-hippocampus and parietal cortex-striatum.

### 3.4. Neurite Density and Their Relationship to GluCEST in 7-Month-Old Mice

**Figure 6:**
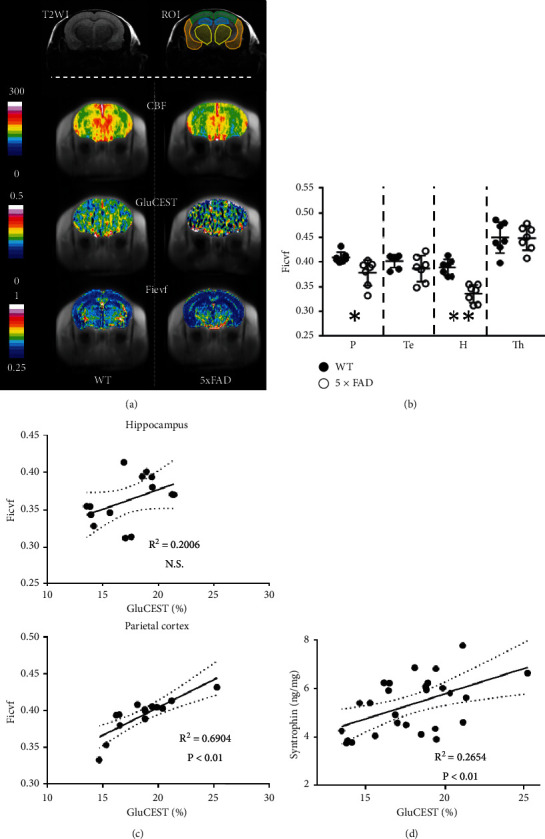
GluCEST effect, Ficvf derived from NODDI, and syntrophin concentration in 7-month-old mice. (a) Representative images of 7-month-old 5xFAD and WT mice showing reduction in both GluCEST effects and Ficvf values in the parietal cortex and hippocampus. (b) ROI Ficvf showing significant reduction in the parietal cortex and hippocampus. (c) Correlation between GluCEST effects and Ficvf showing no correlation in the hippocampus, whereas there is a significant correlation between Ficvf and GluCEST in the parietal cortex. (d) Syntrophin correlation with GluCEST. ^*∗*^*p* > 0.05, ^*∗∗*^*p* > 0.01.

In 7-month-old mice (*n* = 7 for each genotype), Ficvf was calculated from 3-shell DWI images (Figure 6(a)). Ficvf values in 5xFAD mice were significantly decreased in the parietal cortex and hippocampus compared to WT mice (Figure 6(b)). To assess the correlations between Ficvf values and GluCEST effects, linear regressions in these two regions were calculated. There was no correlation in the hippocampus, whereas the parietal cortex revealed a correlation between Ficvf values and GluCEST effects (Figure 6(c)).

### 3.5. Neuronal Density in Hippocampus and Relationship between GluCEST Effect and Synaptophysin

There was no statistical difference in neuronal density between WT and 5xFAD mice (Supplementary Figure 1(a)–1(c)). Synaptophysin in 5xFAD mice was significantly reduced in the parietal cortex (Supplementary Figure 2(a)); however, there was no significant hippocampal reduction (Supplementary Figure 2(b)). All measured synaptophysin correlated to GluCEST effects (Figure 6(d)). Synaptophysin in the parietal cortex and the hippocampus also correlated to GluCEST effects (Supplementary Figure 2(c) and 2(d)).

## 4. Discussion

The results of this study showed that GluCEST effects in 5xFAD mice were regionally decreased in mice as young as four months of age. These results are consistent with those of a proton MRS study where a reduced glutamate signal was found in the hippocampus of 5-month-old 5xFAD mice [36], in which regional GluCEST effects were well correlated with glutamine/creatine ratios measured by proton MRS [19]. In addition, a biological study demonstrated that synaptic degeneration, reflected in the levels of presynaptic synaptophysin [24], begins by four months of age. Given these findings, part of the decreased GluCEST effects may reflect glutamatergic synapse degeneration and loss. In the 5xFAD mice we employed, synaptophysin levels in the whole brain began to decline by four months [36], consistent with our study which showed significant decreases in GluCEST effects both in the parietal and temporal cortex and the hippocampus by four months of age. Brain glutamate concentrations also influenced de novo glutamate synthesis from glucose. At six months of age in an APPswe-PS1dE9 AD mouse model, the initial synthesis rate was reported to be lower than in WT mice, but the equilibrium state of AD mice is identical to that of WT mice [37]. These results suggested that the influence of de novo glutamate synthesis likely exists, but subtly so.

Among the various CBF measurements, the arterial spin labeling (ASL) MRI [38] used in this study is a versatile, noninvasive method that does not require the use of a tracer [39]. A report showing that ASL can visualize distinct regional hypoperfusion in the human preclinical AD brain [40] demonstrated that ASL may detect regional CBF changes linked to early pathological changes in AD. CBF regulation is reportedly reflecting a part of brain neuronal activities [41, 42] tightly coupled to the control of basal levels of cellular metabolism [43, 44], as well as neuromodulatory pathways driven by other neurotransmitters, such as acetylcholine or noradrenaline [41]. Alterations in neuronal activity may be involved in the pathophysiology of AD [45]. In addition, it is reported that resting-state CBF positively correlates with synaptic density [41]. In this study, CBF was reduced following GluCEST effects decrease in 5xFAD mice, and both parameters were well correlated.

In some brain regions, namely, the parietal cortex, the temporal cortex, and the hippocampus, a significant decrease in GluCEST effects at four months of age preceded hypoperfusion. In a human ^1^H-MRS study using 7T MRI, reduced hippocampal glutamate is reported in cases with mild cognitive impairment (MCI) [46]. On the other hand, not only hippocampal CBF is retained in MCI, but some studies also reported an increase in CBF in the left hippocampus [47, 48]. While the mechanisms underlying these results are yet to be elucidated, they may partly be explained by aberrant neuronal excitability [2, 49] or functional compensation [47, 50], both prominent in early AD phases. First, pathologically elevated *Aβ*, especially its oligomeric form, is reported to downregulate A-type K^+^ currents [51] and block neuronal glutamate uptake at synapses [52], leading to aberrant neuronal excitability [49, 53], epileptiform discharge [54], and CBF increase [55]. This mechanism can conserve CBF in 5xFAD mice in early stages, since early adult stage (4 to 10 months) 5xFAD mice are also prone to show epileptiform discharge even without epileptic convulsions [56]. Second, regional hyperutilization of glucose, the major energy resource for the brain, was observed in *Aβ* overexpressed mice [57] including 5xFAD mice [58] in the early phase with subsequent reduction. Since hyperglucose utilization raises CBF in a linear manner [59], CBF reduction may be compensated with hyperglucose utilization in early life stages. These authors [57, 58] assumed that the cause of regional hyperglucose utilization could be associated with compensatory activation of nonaffected neurons against disturbed neuronal circuits. However, the detailed mechanism of the compensation has not been elucidated. Alternatively, glial activation before *Aβ* deposition in 5xFAD mice [24] can also increase glucose utilization [60]. These mechanisms may partly compensate for the CBF reduction in regions with decreased GluCEST effects observed in 4-month-old mice. Therefore, noninvasive detection of early phase regional GluCEST reduction can be employed as an early biomarker for neurodegenerative disease.

The correlation between GluCEST and CBF was statistically different in cortices compared to basal brain regions. While the underlying differences behind these mechanisms could be explained by regional differences regarding the pathological effects of basal CBF on neuromodulatory pathways, they are not yet entirely clear. Both the cholinergic and noradrenergic systems work as CBF neuromodulatory pathways and are impaired in early AD phases [61, 62]. Cortices are more sensitive to the cholinergic system's stimulation-induced CBF changes than basal brain regions [63]. In 5xFAD mice, cholinergic fibers are sequentially impaired in the amygdala, cortex, hippocampus, and basal forebrain by nine months of age [64]. In addition, noradrenergic brain modulation reveals focal CBF changes. Stimulating the locus coeruleus, the principal site of norepinephrine synthesis in the brain and showing dysfunction in early AD phases [62], reflect CBF reductions in deep brain regions but not in cortices [65].

In 7-month-old 5xFAD mice, Ficvf values derived from NODDI reflect dendrite and axon density per unit volume [26]. Pathological changes in AD are associated with dendritic/axonal degeneration followed by neuronal loss [66], suggesting that lower Ficvf values reflect dendritic/axonal degeneration and/or neuronal loss. Significant decreases in Ficvf in the parietal cortex and hippocampus were observed in our measurements. Ficvf in the parietal cortex was correlated with GluCEST effects in parietal cortex, but not in the hippocampus. Neuronal bodies in the hippocampus were preserved in the study by Yang et al., which may be due to differences in pathological changes between the two regions. In AD model mice, A*β* itself was reported to depress synaptic activity before synaptic degeneration [67]. Next, synaptic loss and axonal swelling were observed in the cortex and hippocampus [24]. However, neuronal degeneration [68] and neuronal loss in layer 5 of the cortex preceded synaptic loss and axonal swelling in the hippocampus [69]. Since synaptic density is influenced by sum of measuring synaptic density, axonal/dendritic density, and neuronal density, these reports may partly explain the correlation between Ficvf and GluCEST effects in the parietal cortex, where neuronal loss plays a more important role than in the hippocampus.

Synaptophysin in *ex vivo* specimens which is thought to represent synaptic density correlated with GluCEST effect. This result can be a proof that part of the GluCEST effect can represent synaptic density when the glutamatergic biosynthetic cycle is relatively stable and does not undergo any rapid metabolic changes. However, the GluCEST effect itself may also reflect neuronal activity. In an *in vivo* functional study, decreases in synaptic density in AD represented decreased neuronal activity, partly reflected in the gamma band activity of electroencephalograms or amplitude of low-frequency fluctuations (ALIFFs) from resting-state functional MRI data [45, 70]. ALIFF was reported to be correlated with glutamate concentrations measured by proton MRS in the frontal cortex of women with major depression [71].

The present study has some limitations. GluCEST can introduce some confounding factors in imaging regional distribution of glutamate. GluCEST effects have some pH dependency and are enhanced in acidic conditions [19]. In AD brains, a subtle pH shift was reported to exist [72, 73]. According to these reports, the effect to GluCEST% should be <0.5% according to Figure 1 in [19]. Potential contribution of other brain metabolites or magnetization transfer (MT) background can also influence GluCEST effects. Cai et al. estimated that a 5% maximum confounding factor in GluCEST% can exist in our measurement conditions, but still confirmed the linear correlation with glutamate concentration measured with ^1^H-MRS and GluCEST effects in the rat brain [19]. Our GluCEST% is also correlated with syntrophin concentration which represents synaptic spine density. With respect to the animal model, 5xFAD mice do not show the tauopathy observed in human AD, which also evokes synaptic loss and lessens GluCEST effect [20]. Caution is needed before translating the results of our study to human AD.

## 5. Conclusion

GluCEST MRI has the advantage of not requiring an extrinsic tracer or contrast agent to visualize glutamate brain distributions. While longitudinal studies of both GluCEST and CBF changes in 5xFAD mice revealed that GluCEST effects correlated with regional CBF, reduced GluCEST effects preceded hypoperfusion in part of the cortex and hippocampus, where prominent pathophysiological changes are observed in AD. GluCEST MRI partly reflected changes in synaptic density and activity in an AD mouse model. Altogether, GluCEST imaging may be a viable imaging method for diagnosis and biomarker-based assessment of AD.

## Data Availability

Data and program scripts used to support the findings of this study are available from the corresponding author upon reasonable request.

## Supplementary Materials

Supplementary Materials The supplementary materials represent neuronal density in the hippocampus and regional syntrophin concentration and their relationship to GluCEST effect.
