# Supplementary material for: Longitudinal GluCEST MRI Changes and Cerebral Blood Flow in 5xFAD Mice
Source: Contrast Media Mol Imaging. 2020 Nov 25;2020:8831936. doi: 10.1155/2020/8831936 (PMC7714610; doi:10.1155/2020/8831936)

Supplemental Materials

Longitudinal changes in gluCEST MRI and their relationship to cerebral blood flow in a 5xFAD mouse

Hironaka Igarashi, Satoshi Ueki, Hiroki Kitaura*, Tae Kera, Ken Ohno, Masaki Ohkubo§, Mika Terumitsu-Tsujita, Akiyoshi Kakita*, Kwee L Ingrid†

Center for Integrated Human Brain Science, Brain Research Institute,

University of Niigata, Niigata, Japan

* Department of Pathology, Brain Research Institute, University of Niigata, Niigata, Japan

§ Department of Radiological Technology, School of Health Sciences, Faculty of Medicine, University of Niigata, Niigata, Japan

† Neurology, University of California, Davis, USA

Figure S-1

Neuronal density in hippocampus

Nissle stain specimens of (A) WT CA1 region of hippocampus and (B) 5xFAD.

Neuronal numbers of both mice were identical.


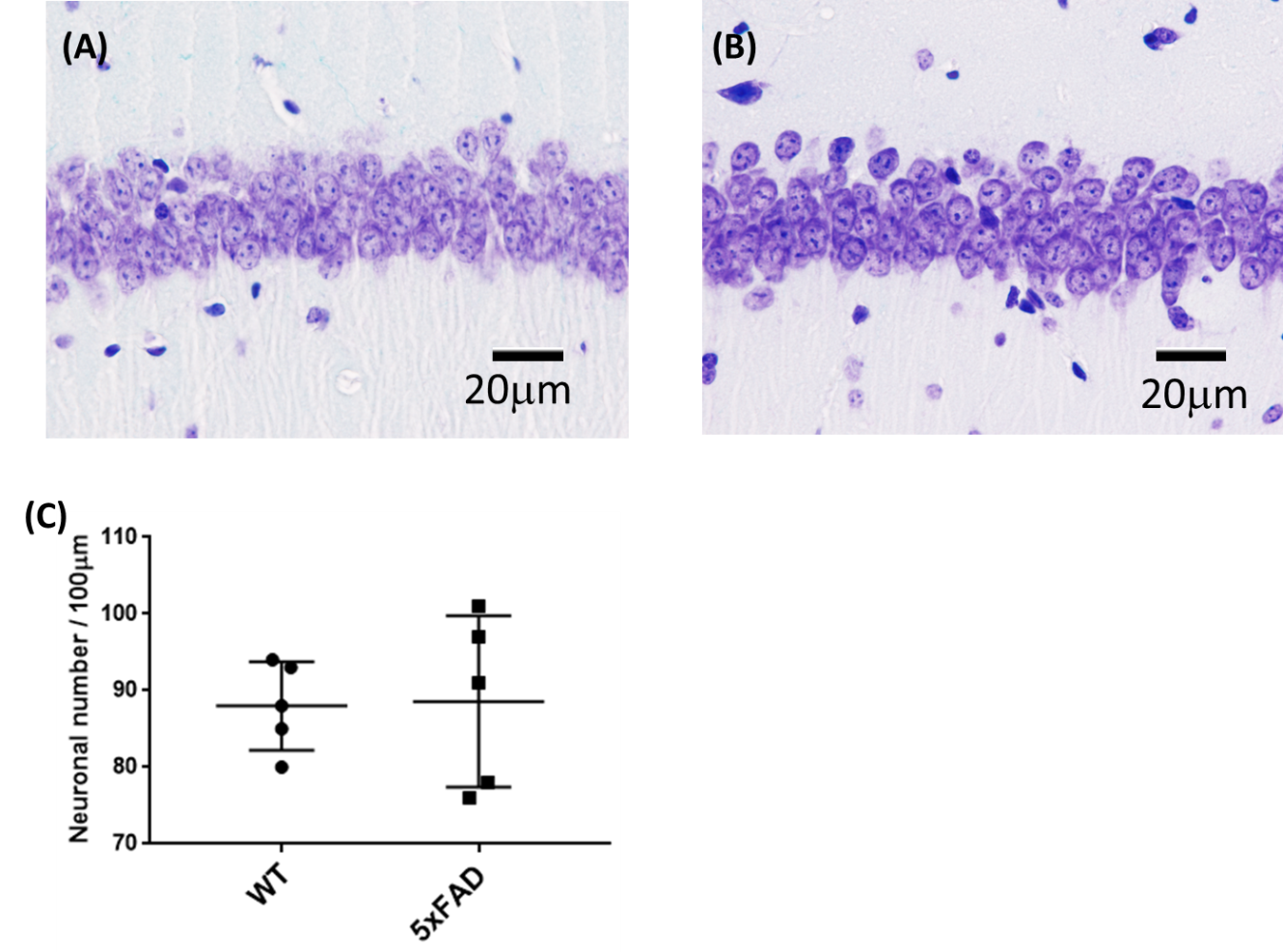


Figure S-2.

Regional syntophin concentration and their relationship to gluCEST effect.

Synaptophysin of 5xFAD mice was significantly (A) reduced in parietal cortex, but (B) reduction in hippocampus of 5xFAD mice was not significant. (C,D) Synaptophisin in both parietal cortex and hippocampus also correlated to gluCEST effect.


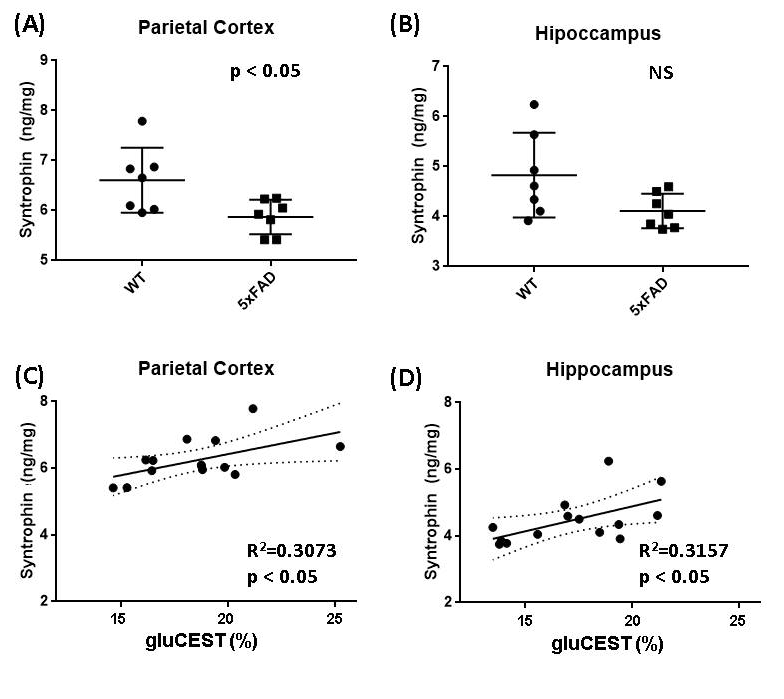

Supplement: Supplementary Materials — The supplementary materials represent neuronal density in the hippocampus and regional syntrophin concentration and their relationship to GluCEST effect. [file 8831936.f1.docx]
